# Case Report: Development of Diffuse Large B Cell Lymphoma a Long Time After Hairy Cell Leukemia

**DOI:** 10.3389/pore.2022.1610338

**Published:** 2022-04-29

**Authors:** Zsófia F. Nagy, Kata Ferenczi, Ildikó Istenes, Hanna Eid, Csaba Bödör, Botond Timár, Judit Demeter

**Affiliations:** ^1^ Department of Internal Medicine and Oncology, Semmelweis University, Budapest, Hungary; ^2^ HCEMM-SE Molecular Oncohematology Research Group, 1st Department of Pathology and Experimental Cancer Research, Semmelweis University, Budapest, Hungary

**Keywords:** hairy cell leukaemia, non-Hodgkin lymphoma, diffuse large B cell lymphoma, transformation, case report

## Abstract

Hairy cell leukaemia (HCL) is a rare B cell malignancy with an indolent course leading to pancytopaenia due to bone marrow infiltration. It has been proposed that HCL patients are at risk of developing a secondary malignancy, with a marked likelihood of the development of other hematologic malignancies including Hodgkin lymphoma and high-grade non-Hodgkin lymphomas. Here, we present the case of two patients who developed diffuse large B cell lymphoma after a long course of hairy cell leukaemia. In the case of the female patient, we report on the occurrence of a third malignant disease, which is very uncommon. With our case descriptions we contribute to the very small number of similar cases reported.

## Introduction

Hairy cell leukaemia (HCL) accounts for approximately 2% of all leukaemias. Its characteristics include nearly mature B-cells with cytoplasmic projections (hairy appearing) in the blood and bone marrow, which show a distinct immunophenotype (abnormally large cells, positive for the following markers: CD19, CD20, CD22, CD11c, CD25, CD103, and FMC7). Cytopaenias and splenomegaly are a common sign of HCL as well, while peripheral lymphadenopathy is rare among HCL patients (1). The diagnosis of HCL is based on microscopic and flow cytometry examination of the peripheral blood. For the unequivocal diagnosis of HCL the histological examination of a bone marrow biopsy is inevitable (2). The disease is mostly slowly progressive, and has a favorable outcome when treated appropriately (3).

In 2011, it has been discovered that the vast majority of classical HCL cases carry the p.V600E driver mutation in the *BRAF* oncogene (4). BRAF inhibitors are now an effective treatment option for certain *BRAF* positive patients who are refractory to purine analogs used in first-line (clabribine or pentostatin) and immunotherapeutic drugs used second-line (rituximab) (5). Vemurafenib may be used as monotherapy or in combination with rituximab in refractory or in relapsed HCL patients (6). Vemurafenib itself is the treatment of choice in the case of granulocytopenic HCL patients suffering from an infection (2).

Based on epidemiological data, HCL patients are at risk of secondary cancers. Among HCL patients, infections and their complications are responsible for the majority of mortalities, while these malignancies are the second most common causes of death (7). Zheng et al have reported an increased risk of developing non-Hodgkin lymphomas among HCL patients (8).

The literature on HCL transforming into diffuse large B cell lymphoma (DLBCL) is sparse. Several studies aimed to reveal an association between these two different entities. The question whether it is a transformation or an unlucky cooccurrence of the two malignancies remains debated (9). Hereby we would like to contribute to the elucidation of the issue by reporting the case of two HCL patients who both developed DLBCL after a long history of a non-symptomatic stable hairy cell disease.

## Case Report

Patient 1 was a 47-year-old woman when she first presented at a medical provider in 1997 with pain in the upper left quadrant of the abdomen. In the anamnesis several episodes of meningitis (during her childhood) were reported. Due to hearing loss, headaches, and ear pain a mastoidectomy was performed on her. She was also diagnosed with epilepsy.

Physical examination revealed splenomegaly. Splenectomy was performed which secured the diagnosis of typical hairy cell leukemia. Since the involvement of the *BRAF* gene was not known at the time of diagnosis, the study of the genetic background of HCL had not been performed in her case. This disease was stable from a haematological point of view for another 8 years. Over the years she developed symptoms of peripheral arterial disease and complained of progressive dysbasia (difficulty of walking), thus an iliacal stent implantation was carried out in 2003 which had to be reoperated due to stenosis the following year.

In March of 2005, at the age of 55 she complained of stomach pain, weight loss, melaena and haematemesis. On gastroscopy, an exulcerated and bleeding gastric tumor was diagnosed. Due to the ongoing bleeding biopsy was not taken at that time from the mass. Upon CT (computed tomography) scans infiltration of the left adrenal gland was seen. Finally, in July of the same year a total gastrectomy and oesophago-jejunostomy was carried out. On histological examination of the specimen the diagnosis of DLBCL with centroblastic morphology and with non-germinal center (non-GC) B-cell phenotype (CD20 positive, MUM1 positive, CD10 negative, Ki 67 100%) was made. Unfortunately, no residual sample remained after the diagnostic procedure for further investigations. She was referred to our department. The bone marrow biopsy showed normocellular marrow with no signs of lymphoma infiltration and with less than 1% CD20^+^ B-cells. On staging examinations mediastinal, axillary, retroperitoneal lymphadenomegaly and a mass in the left adrenal region and around the head of the pancreas were described. R-CHOP (rituximab, cyclophosphamide, doxorubicin, vincristine, prednisolone) immunochemotherapy was started. After 2 cycles of the therapy a remarkable regression was seen on CT scans. She successfully completed 8 cycles of R-CHOP therapy. The final CT examinations also showed complete remission.

Due to the pressure applied to the left kidney by the enlarged lymph nodes in the retroperitoneal region, the parenchyma became narrower, while the pyelon dilated. According to ultrasound results the obstruction was located directly on the pyelouretheral junction. A transrenal drainage of the urine was initially required due to the kidney dysfunction. Several additional urological interventions were carried out to restore the function of the kidney, but as a complication of chronic pyelonephritis, the condition slowly progressed into end stage kidney disease. The patient has undergone nephrectomy in 2010.

**FIGURE 1:**
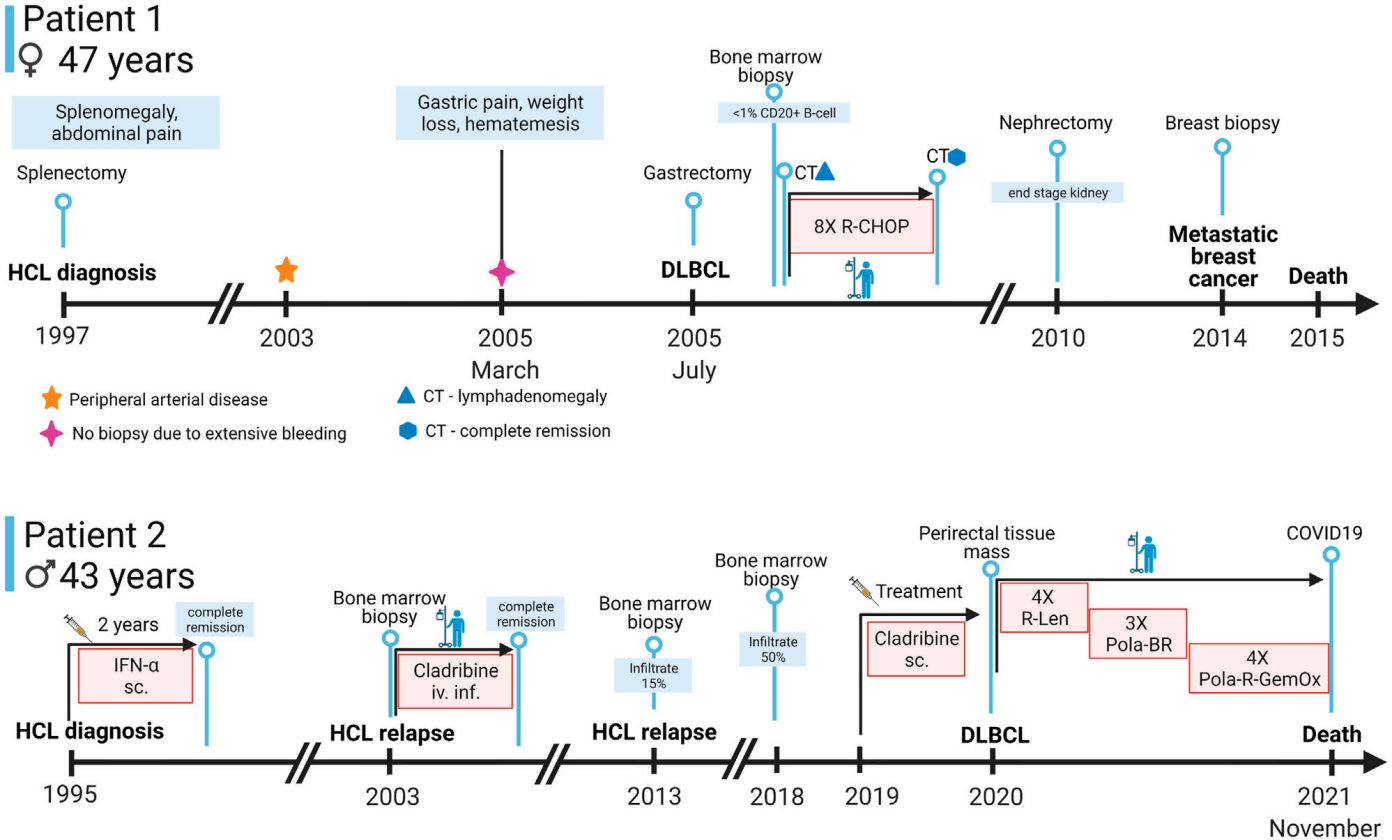
Timeline of symptoms, diagnostic approaches, and treatments of the patients. Abbreviations: sc., subcutaneous; iv.inf., intravenous infusion; CT, computer tomography; IFN-α, interferon alpha; R-Len, Rituximab-Lenalidomide; Pola-BR, polatuzumab vedotin-bendamustin-rituximab, Pola-R-GemOx, Polatuzumab vedotin-rituximab-gemcitabine-oxaliplatin (Created with BioRender.com).

In the summer of 2014, she complained of pain in her bones around the sacral region. On physical examination a coin sized mass was palpated in the upper outer quadrant of the left breast. Aspiration cytology of the lump showed the spread of a CK7 positive poorly differentiated high grade tumor to one of the regional lymph nodes. PET-CT scans showed two fluorodeoxyglucose (FDG) avid masses in the left breast. Furthermore, metastases were present in almost all imaged bones, in the lungs, in the liver, in the right adrenal gland and in several regions of the skin. Enlarged lymph nodes with abnormal metabolism were observed above and under the diaphragm. Sadly, the patient passed away due to her third malignancy, a metastatic breast cancer, in 2015 at the age of 65 (Figure 1, Patient 1).

Patient 2 was a 43-year-old man at the time of HCL diagnosis in 1995 with type 2 diabetes mellitus (treated with oral antidiabetic drugs), peripheral neuropathy, cardioverter implantation, gastrointestinal polypectomy, gastro-oesophageal reflux disease, gout and deep vein thrombosis in the past medical history.

He received alpha interferon treatment in the first 2 years after the diagnosis which resulted in a complete clinical remission. In July of 2003, at the age of 51, his disease relapsed and the patient was referred to our department. We treated the patient with a course of intravenous cladribine, which led to complete clinical remission again. The patient presented regularly for check-up examinations at our department and showed stable normal blood cell counts, with no detectable cells with hairy cell phenotype on the flow cytometric examination of the peripheral blood.

In 2013, relapse of HCL was suspected due to a slight decrease in the patient’s cell numbers. Bone marrow biopsy was performed, which revealed a moderate (15% aberrant B-cells) infiltrate of HCL.

**FIGURE 2:**
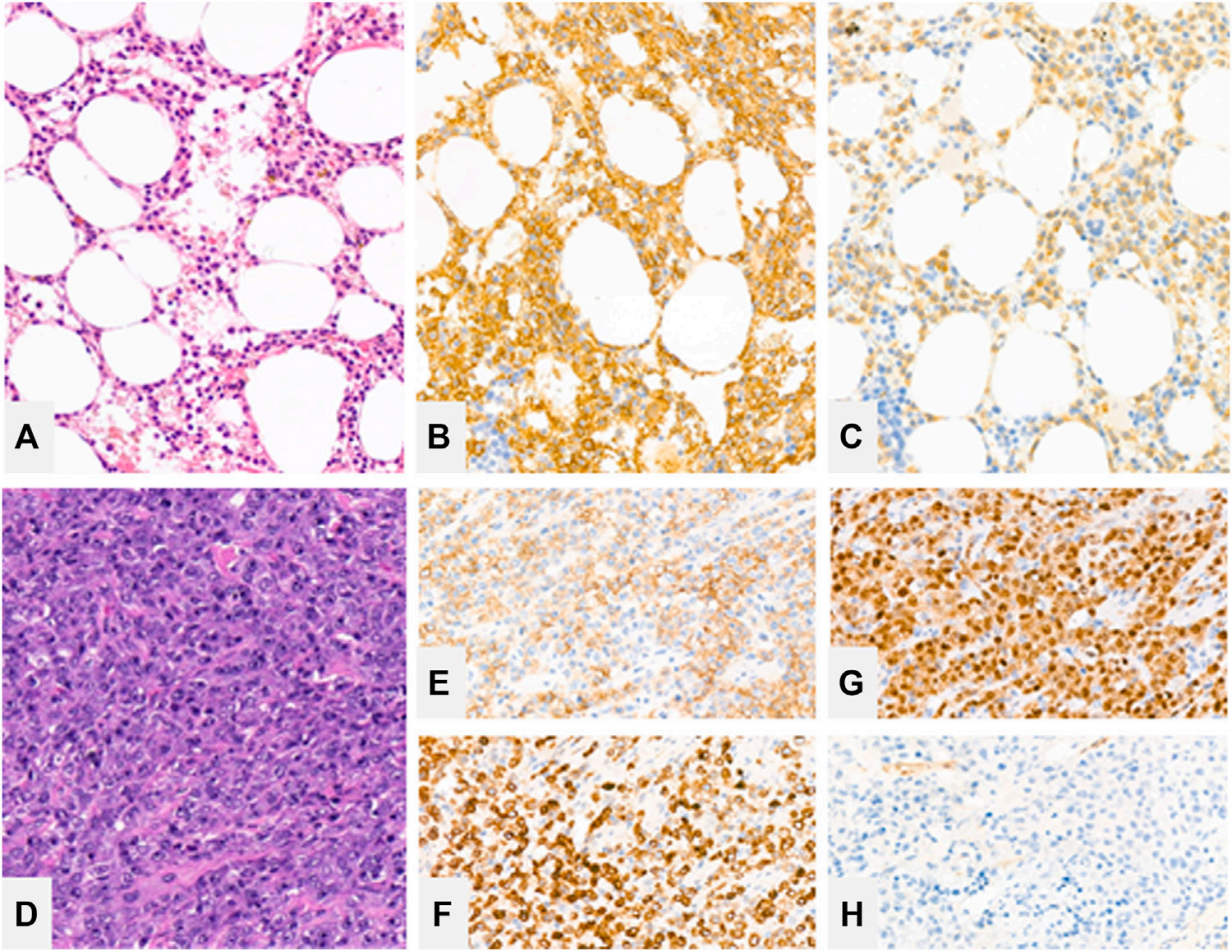
Bone marrow trephine biopsy showing extensive marrow engagement by hairy cell leukemia with a predominantly interstitial pattern [**(A)**—hematoxylin and eosin (H&E), ×20 magnification], highlighted by CD20 **(B)** and BRAF **(C)** immunohistochemistry. Rectal resection biopsy showing large immunoblastic cells [**(D)**—H&E, ×20] with a dim CD20 expression **(E)** with strong expression of CD79a **(F)** and MUM1 **(G)**. Cells lack CD10 expression **(H)**.

The patient’s granulocyte count continued to decline after years of watchful follow up, so another bone marrow biopsy was performed in November of 2018. The bone marrow was severely infiltrated with typical (CD11c+, CD103+, CD25+, CD19+, CD20+) B-cells as shown by flow cytometry of the aspirate. The bone marrow trephine biopsy also confirmed the extensive marrow infiltration by hairy cell leukemia with a predominantly interstitial pattern (Figure 2A) proven by CD20 (Dako, Glostrup, Denmark) and BRAF (VE1, Ventana Medical Systems, Tucson, AZ) immunohistochemistry (Figures 2B,C). From January of 2019 the patient received subcutaneous cladribine treatment. From April of 2019 the treatment was complemented with eight cycles of rituximab, each time 375 mg/m^2^ in the form of intravenous infusions without complications.

In 2019 the patient complained of episodes of fever of unknown origin. In order to rule out another relapse of the disease, a bone marrow biopsy was carried out again, which showed normal hematopoeisis, without signs of a relapse. In the background of the fever a perirectal abscess was discovered. The patient developed similar perirectal abscesses five times over the course of a half year. Some of these abscesses formed fistules and drained in the direction of the neighboring organs with the wounds showing limited healing capacity. In February of 2020, an abscess in the perirectal region was evacuated and resected and the patient has undergone a rectal exstirpation at the same time.

Histological examination of the resected abscess and its surroundings showed large transformed lymphoid cells in a necrotic background. Morphologically the transformed cells were similar to immunoblasts and had prominent inclusion-like nucleoli. The cells displayed CD20 positivity, CD79a (Dako, Glostrup, Denmark) positivity with MUM1 (Dako, Glostrup, Denmark) expression lacking CD10 (Leica-NovoCastra, Newcastle, UK) expression (Figures 2D–H). The tumor cells were highly and uniformly reactive with Bcl-2 (not shown) staining. However, c-myc (not shown) staining was present in only around 30% of the investigated cells. Based on these the diagnosis of diffuse large B cell lymphoma with non-GC B cell phenotype was established. Due to the ongoing inflammatory process in the area, the perineum could not be accurately assessed with PET-CT. Lymph nodes and extranodal regions were not affected by the lymphoma.

In the state of severe immunosuppression caused by the rectal abscess and following the rectal extirpation, the patient was not fit enough to receive classical immunochemotherapy for his DLBCL, but received four cycles of rituximab and lenalidomide (4X R-Len) combination treatment according to Zinzani’s protocol (10) accompanied by preventive LMWH resulting in gradual clinical improvement. By August of 2020 both the rectoprostatic and the rectourethral fistules closed and the patient’s micturition returned to normal. The PET-CT examination performed after the second cycle showed a less widespread FDG avid process. After the fourth cycle of rituximab–lenalidomide therapy, the patient complained of dyspnoea which was present even upon minimal exertion. CT angiography revealed an extensive pulmonary embolism which originated from the partially old and partially novel deep vein thrombosis of the left superficial femoral vein.

From July of 2020, his treatment was switched to a combination of polatuzumab-rituximab-bendamustine (Pola-BR). His vitals and overall state were stable. A restaging PET-CT after the third cycle of treatment was carried out, which only showed partial remission, thus the patient’s treatment was further escalated.

After the 3 cycles Pola-BR, a protocol consisting of polatuzumab, rituximab, gemcitabine and oxaliplatin (Pola-R-GemOx) was implemented. A restaging interim PET-CT after the third cycle of Pola-R-GemOx showed good response. As a side effect of the fourth cycle of therapy (4X Pola-R-Gemox), the patient felt weak, complained of vertigo and lost his appetite. These adverse effects could not be tolerated, so the patient refused to accept the last, 5th planned cycle of the therapy. Nevertheless, the closing PET-CT examination carried out in July of 2021 showed complete metabolic remission of the lymphoproliferative disease.

He was admitted to the hospital in late November of 2021 for a parastomal hernia operation and for the closure of the urethroperineal fistule. A perianal negative pressure wound therapy was implemented and an epicystostomy was formed. The postoperative days were uneventful. However, 2 days later the patient had to be readmitted because of shortness of breath. He tested positive for COVID-19 and developed a rapidly progressing pneumonia and even though the patient received active antiviral and supportive therapy, his oxygen demand increased and he passed away due to the complications of the COVID-19 infection a month after his admission. No autopsy was performed (Figure 1, Patient 2).

## Discussion

Hairy cell leukemia is a disease with a fairly good prognosis if the patient receives adequate treatment. Over the course of their follow-up, approximately 10% of HCL patients develop secondary malignant diseases. These malignant diseases and infections might be held accountable for a significant proportion of mortality among all HCL patients. The three most common types of secondary tumors among observed HCL patients were Hodgkin lymphoma, non-Hodgkin lymphomas and thyroid cancer (11).

The male patient in our report was diagnosed with DLBCL 25 years after the initial HCL diagnosis, whilst in the case of our female patient 8 years have passed between the diagnoses. It has been described in the literature based on the case of eight patients that 178–395 months pass on average between the diagnosis of HCL and DLBCL (12). It is particularly interesting, that the female patient even developed a third tumor, according to reports it happens in only about 0.9% of HCL patients (11). We might attribute the development of breast cancer in the female patient to the fact that one in every seven women develops breast cancer in her lifetime (13). Since there is no description in the literature about breast cancer being the third tumor of a HCL patient, we may classify it as a concomitant event.

Whether HCL patients are more susceptible for secondary malignancies is still debated, many studies with contradicting results have been published (14–16). The question arises, what makes HCL patients more prone to these tumors than others. The role of purine nucleoside analogs has been proposed, but the ongoing debate has not reached consensus yet. Rosenberg et al. could not identify an increased risk of secondary tumors among young HCL patients who have been treated with cladribine (12). In case of the female patient presented above, the adverse effects of HCL related medications can be certainly excluded, since the patient has undergone splenectomy for her HCL as the sole treatment, no drugs were administered.

Another reason behind the increased cumulative incidence of malignant diseases in HCL patients could be due to their advanced age or other environmental or lifestyle risk factors of cancers. Tendency has it, that the longer the survival after the HCL diagnosis, the greater the risk for developing secondary tumors (17). This is another argument for the involvement of additional factors in the development of the secondary malignancies.

Limitations of our work include lack of residual tissue sample from the case of Patient 1 and the unsuccessful retrieval of the second sample of Patient 2. Therefore, we could not perform additional molecular testing that would clarify the clonal relatedness of these samples. During our more than 40 years, we have been taking care of HCL patients in our institution, only two instances were observed, when HCL patients also developed DLBCL.

This proves the need to further investigate the possibility of a HCL transforming into a high-grade lymphoma. A secondary hematological malignancy might be overlooked and wrongly interpreted as the recurrence of HCL. With our interesting case report, we wish to contribute to the infinitesimal number of cases published in the field. Furthermore, we would like to raise awareness among physicians about this rare but not omissible possibility of diffuse large B cell lymphoma, that may develop in patients a long time after the successful treatment of HCL.

## Data Availability

The original contributions presented in the study are included in the article/Supplementary Material, further inquiries can be directed to the corresponding author.
